# Treatment of Benign Pigmented Lesions Using Lasers: A Scoping Review

**DOI:** 10.3390/jcm14113985

**Published:** 2025-06-05

**Authors:** Aurore D. Zhang, Janelle Clovie, Michelle Lazar, Neelam A. Vashi

**Affiliations:** 1 Department of Dermatology, Boston University School of Medicine, 609 Albany St., J502, Boston, MA 02118, USA; 2 Dermatology Institute of Boston, Boston, MA 02118, USA

**Keywords:** laser, melanocytic lesions, café-au-lait macules, Becker’s nevus, nevus spilus, nevus of Ota

## Abstract

Lasers are widely employed in the treatment of melanocytic lesions. This scoping review evaluates 77 studies on the efficacy and safety of laser treatments for café-au-lait macules (CALMs), nevus of Ota (NOA), Becker’s nevus (BN), lichen planus pigmentosus (LPP), and other pigmented lesions. The Q-switched neodymium-doped yttrium aluminum garnet (Nd:YAG), particularly the 1064 nm, is the most frequently utilized laser, demonstrating strong efficacy for NOA and other dermal pigmentary disorders. Medium-wavelength lasers, including the Q-switched ruby and Alexandrite lasers, also show promise, though results vary based on lesion depth, skin type, and treatment protocols. Recurrence and adverse effects, including post-inflammatory hyperpigmentation (PIH) and hypopigmentation, are common, particularly in patients with darker skin tones. Future studies should standardize and optimize laser parameters across lesion types and skin tones, improve long-term efficacy, and prioritize inclusion of patients with diverse Fitzpatrick skin types to evaluate differential outcomes and promote equitable treatment efficacy.

## 1. Introduction

Lasers have emerged as an indispensable tool in dermatologic treatment, offering precision and versatility in managing a wide array of skin conditions, including melanocytic lesions [1]. Melanocytic lesions range from common conditions such as ephelides and lentigines to more complex entities like nevus of Ota and lichen planus pigmentosus [2]. The evolution of laser technologies has enabled clinicians to treat these lesions with greater specificity and minimal adverse side effects [2,3].

The application of lasers in melanocytic lesions works through selective photothermolysis, wherein pigment chromophores in skin absorb light at specific wavelengths, resulting in targeted destruction of melanin without damage to the surrounding tissue [4]. Selection of specific laser parameters such as wavelength, pulse width, and energy level allows for accurate targeting of specific chromophores, and use of the most appropriate laser for treatment [5]. Wavelength selectivity restricts absorption to affect only the targeted chromophore [5]. Laser pulse width is selected to be less than or equal to the target chromophore’s thermal relaxation time, to keep thermal damage limited to the undesired pigment. Laser energy levels are selected based on desired end point [5]. Commonly utilized lasers include the Q-switched lasers, such as the Q-switched neodymium-doped yttrium aluminum garnet (Nd:YAG), ruby lasers, and Alexandrite lasers, which are effective in treating dermal and epidermal pigmentary disorders [5]. IPL lasers are most effective in treating epidermal pigmented lesions, and fractional lasers use ablative and non-ablative resurfacing technology. Overall, response to laser treatment is influenced by a myriad of factors, necessitating a personalized approach [2,6].

While congenital melanocytic nevi are commonly treated with lasers, there is a paucity of data regarding efficacy across melanocytic lesions. Lesion such as nevus of Ota (NOA), lichen planus pigmentosus (LPP), Becker’s nevus (BN), and Hori’s nevus present unique therapeutic challenges due to the varying degrees of dermal and epidermal involvement [7,8,9,10]. The most common post-laser complications in the treatment of these pigmented lesions include post-inflammatory hyperpigmentation (PIH), hypopigmentation, and erythema; therefore, treatment efficacy must be carefully balanced against the risk of adverse effects [7,8,9,10]. Despite promising clinical outcomes reported in individual studies on lasers, the variance in treatment protocols, patient populations, and follow-up durations underscores the need for a comprehensive synthesis of the evidence.

This scoping review aims to evaluate the efficacy, periprocedural management, and side effect profiles of lasers in the treatment of melanocytic lesions, excluding congenital nevi. Congenital nevi were excluded from this study as they have a distinct pathophysiology. By systematically analyzing the available literature, we seek to identify patterns of clinical response, common adverse effects, and overall efficacy to provide clinicians with evidence-based guidance to optimize outcomes for patients with these complex, and often challenging, pigmentary disorders.

## 2. Materials and Methods

In accordance with the Preferred Reporting Items for Systematic Reviews and Meta-Analyses (PRISMA) guidelines, MEDLINE, EMBASE, and PubMed databases were systematically reviewed in December 2024 [11]. The following search phrases were utilized: “laser dermal melanosis”, “Becker’s nevi laser”, “nevi of Ota and Ito laser”, “speckled lentiginous nevi laser”, “nevus spilus laser”, “dermal melanocytosis laser”, and “segmental pigmentation disorder laser”. This search yielded 1717 articles. EMBASE was then searched with the same search criteria, and after duplicate records were removed, 325 additional articles were identified. Cochrane was then cross-referenced with the search criteria of “laser skin”, “laser melanin”, and “laser”. No additional articles were found through a cross reference of the Cochrane database. In total, 1768 articles were selected for further screening.

**Figure 1:**
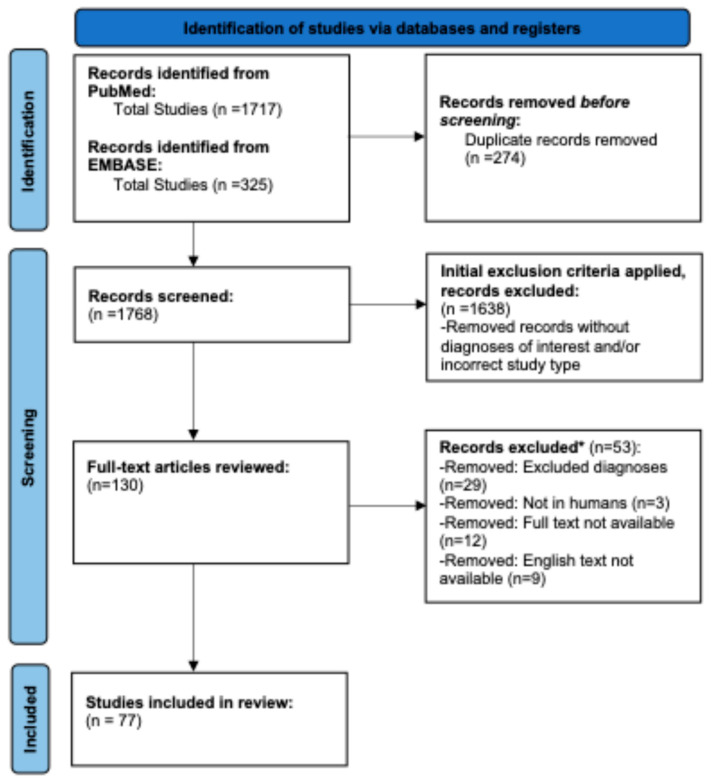
PRISMA flowchart: outline of extracted data, presented as a PRISMA flowchart. * All records were reviewed by authors A.D.Z. and M.L. who cross-referenced selected articles to ensure data extraction was equivalent across individuals.

Initial exclusion criteria were applied, eliminating articles that lacked predetermined diagnoses, as well as case reports and epidemiologic studies. This resulted in 130 full articles. Each entry was then inputted into a shared Google Sheet, and PubMed IDs were utilized to identify papers when available; otherwise, the DOI or full citation was used. Authors A.D.Z. and M.L. reviewed the full text for all 130 articles and removed all articles with excluded diagnoses, non-human studies, and those where the full text was unavailable or not available in English. All records were reviewed by authors A.D.Z. and M.L., who cross-referenced selected articles to ensure data extraction was equivalent across individuals. After this analysis, there were 77 articles included (Figure 1) [12,13,14,15,16,17,18,19,20,21,22,23,24,25,26,27,28,29,30,31,32,33,34,35,36,37,38,39,40,41,42,43,44,45,46,47,48,49,50,51,52,53,54,55,56,57,58,59,60,61,62,63,64,65,66,67,68,69,70,71,72,73,74,75,76,77,78,79,80,81,82,83,84,85,86,87]. Successful clearance of lesions within a study was considered if 50% of patients or more reported clearance. An expanded analysis of café-au-lait macules (CALMs), nevus of Ota (NOA), Becker’s nevus (BN), and nevus spilus (NS) was undertaken due to the large proportion of assessed studies these diagnoses comprised, in order to better ascertain unique subgroup characteristics.

## 3. Results

### 3.1. Demographic Data of Included Studies

Studies were conducted most frequently on café-au-lait macules (35 studies), nevus of Ota and acquired bilateral nevus of Ota-like macules and Hori’s nevus (29), Becker’s nevus (20), nevus spilus (12), dermal melanocytosis (4), Rhiel’s melanosis (2), lichen planus pigmentosus (2), nevus of Ito (2), dermal melanocytosis (3), erythema dyschromia perstans (1), erythema follicularis faceie (1), and linear and whorled nevoid hypermelanosis (1). The number of enrolled lesions ranged from 4 to 620 and studies enrolled 42.82 patients on average. The age of patients enrolled ranged from 0 to 77 years. The Nd:YAG was the most studied laser (32 studies), followed by the Q-switched ruby (14) and the Q-switched Alexandrite laser (12). Of all the studies included, only two commented on the cost of treatment and patient opinion of cost. Furthermore, only 20 studies included data on patient preference of treatment.

### 3.2. Café-au-Lait Macules

Thirty-five studies were published on café-au-lait macules (CALMs) treated with lasers. There was an average of 50 patients enrolled in each study. Six studies included patients with Fitzpatrick skin type (FST) I, nine with FST II, thirteen with FST III, fifteen with FST IV, four with FST V, and two with FST VI. In twenty-three studies anesthetics were utilized, either topical or local, prior to laser treatment. The most commonly utilized lasers were the Nd:YAG (17 studies), Alexandrite PicoWay (6), and the Q-switched ruby (6). The average number of treatments for patients ranged from 1 session to 50. Twenty-one studies utilized aftercare, with nine treating patients with topical or antibiotics, two utilized steroids, and two utilized hydroquinone. The most frequently reported side effects in CALMs treated with lasers were hyperpigmentation (15 studies) and hypopigmentation (12) (Table 1).

### 3.3. Nevus of Ota, Acquired Bilateral Nevus of Ota-like Macules, Hori’s Nevus

Twenty-nine studies were published on nevus of Ota (NOA), acquired bilateral nevus of Ota-like macules (ABNOM), or Hori’s nevus. These were grouped for analysis as many studies did not distinguish between them. An average of 37 patients were enrolled in each study. FST I patients were involved in two studies, FST II in four, FST III in eleven, FST IV in sixteen, FST V in ten, and FST VI in four. Nineteen studies utilized topical or local anesthetic prior to treatment. The Nd:YAG was the most studied laser (12 studies), followed by the Q-switched ruby (9), and the PicoWay Alexandrite (7). On average, patients received 5.29 treatments. Fifteen studies utilized aftercare, with eight utilizing topical or oral antibiotics, four steroids, and one study utilized post-laser hydroquinone. The most reported side effects in NOA/ABNOM/Hori’s nevus treated with lasers were hyperpigmentation (13 studies) and erythema (10) (Table 2).

### 3.4. Becker’s Nevus

Twenty studies were conducted on the efficacy of lasers in the treatment of the melanocytic component of Becker’s nevus (BN). An average of 39 patients were enrolled in each study. Regarding FST: one study included patients with FST I, four with FST II, nine with FST III, ten with FST IV, four with FST V, and one with FST VI. Eleven studies utilized topical or local anesthetics prior to treatment. The Nd:YAG was the most studied (five studies) followed by intense pulsed light (IPL, 4). On average, patients received 2.42 treatments. Eleven studies utilized aftercare, with four utilizing topical or oral antibiotics, four utilizing steroids, and only two studies utilized post-laser hydroquinone. The most reported side effects in Becker’s nevus treated with laser were hyperpigmentation (nine studies), erythema (seven), and hypopigmentation (seven) (Table 3).

### 3.5. Nevus Spilus

There were twelve studies conducted on nevus spilus (NS). An average of 46 patients were enrolled. Of the studies who commented on FST, two included patients with FST I, three with FST II, four with FST III, four with FST IV, and one with FST V. Five studies utilized topical or local anesthetic prior to treatment. The Nd:YAG was the most studied laser (five studies) followed by the Alexandrite PicoWay (three). Six studies utilized aftercare, with three utilizing topical or oral antibiotics, two utilizing steroids, and two using post-laser hydroquinone. The most reported side effects in nevus spilus treated with laser were hyperpigmentation (five studies), hypopigmentation (four), erythema (four), and pain (four) (Table 4).

### 3.6. Other Lesions with Less than Five Studies

Three studies utilized lasers in the management of dermal melanocytosis. For dermal melanocytosis, the most commonly utilized lasers were the Nd:YAG (one study), Q-switched ruby (one), Q-switched Alexandrite (one), and Alexandrite PicoWay (one). The side effects noted in dermal melanocytosis were post-inflammatory hyperpigmentation (PIH), edema, erythema, and blistering. Two studies were conducted on the efficacy of laser treatment in Rhiel’s melanosis, with the Nd:YAG (one study) and fractional lasers (one) being the most studied lasers. Noted side effects were hyperpigmentation, erythema, and hypopigmentation. Two studies utilized lasers in the management of nevus of Ito which utilized the Q-switched ruby (one study), Nd:YAG (one), Q-switched Alexandrite (one), and the Alexandrite PicoWay (1). PIH, edema, and erythema were commonly reported side effects. Two studies were conducted on the efficacy of lasers on lichen planus pigmentosus (LPP), both of which utilized the Nd:YAG laser. The side effects noted were PIH, hyperpigmentation, erythema, and hypopigmentation. There was one study conducted on lasers in the management of erythema dyschromia perstans (EDP), one on erythromelanosis follicularis faciei (EFF), and one on linear and whorled nevoid hypermelanosis (LWNH).

## 4. Discussion

Melanocytic lesions can be challenging to treat with topical treatments. Medium-wavelength lasers that penetrate the dermis allow for the clearance of dermal pigment and improved overall efficacy. Across all studies, the Nd:YAG was the most frequently utilized laser in the treatment of benign pigmented lesions, specifically the 1064 nm wavelength one. The Nd:YAG can utilize two wavelengths, 532 nm for more superficial pigments and 1064 nm for deeper dermal pigments, as well as resurfacing and textural concerns. Within dermatology, the Nd:YAG is utilized for a myriad of problems ranging from hair removal to nail psoriasis and more [88,89]. Within the context of benign pigmented lesions, the Nd:YAG was successful in treating CALMs, NOA, ABNOM, Hori’s nevus, BN, and NS across a variety of FSTs. Most commonly, lesions were treated with a 3 mm spot size and a pulse duration of either 10 or 25 nanoseconds. Lasers were typically applied to intact skin without active infection, inflammation, or recent sun exposure, and patients were generally instructed to avoid photosensitizing agents prior to treatment. In addition, studies reported offering or utilizing anesthetic measures, such as topical lidocaine, cooling devices, or cryogen spray, to improve patient comfort during procedures. Fluence and number of sessions varied between studies, and only a minority clearly reported rationale for their selected parameters. While some lasers demonstrated a 100% success rate in the treatment of pigmented lesions, these findings should be interpreted with caution due to the limited number of studies. Small sample sizes increase the risk of overestimating efficacy, particularly in the absence of long-term follow-up or standardized outcome measures.

Café-au-lait macules (CALMs) are common hyperpigmented lesions that can vary greatly in size and degree of pigmentation. They were the most studied benign pigmented lesions, included in 35 studies. Most patients enrolled had FST of III or greater. In the treatment of CALMs, the most utilized lasers were the Nd:YAG, Alexandrite PicoWay, and the Q-switched ruby. Studies reported the successful use of all three lasers in the treatment of CALMs. Other meta-analysis on the treatment of lasers in CALMs have found that the Q-switched Nd:YAG is the most efficacious treatment, though they also note the use of other lasers such as the ruby laser and the Alexandrite laser [90,91]. Treatment of CALMs with lasers is well-studied, as one of the few effective treatment options. These lesions, while benign in nature, can significantly impact QoL. Therefore, if lesions are large or in a cosmetically sensitive location, they are often treated for overall cosmesis. The most reported side effects in CALMs were hyperpigmentation and hypopigmentation. Reported hyperpigmentation is likely in the setting of PIH due to the accumulation of melanin from the destruction of melanocytes in combination with inflammation-related increases in melanin production [92]. PIH is more commonly appreciated in more richly pigmented skin, such as that of FST III and above [93]. Therefore, it is not surprising that the given cohort with a higher proportion of patients with FST III and above commonly reported PIH and hyperpigmentation.

Nevus of Ota (NOA), acquired bilateral nevus of Ota-like macules (ABNOM), or Hori’s nevus (HN) were included in 29 studies. Many studies did not distinguish these diagnoses in their enrollment criteria. Therefore, for the sake of analysis, these were grouped into one category. NOA is a congenital dermal melanocytosis, typically unilateral, hypothesized to be caused by failure of melanocyte migration from the neural crest during embryogenesis, resulting in melanocytes trapped in the dermis most appreciated in the distribution of the trigeminal nerve [94]. ABNOM (also known as HN) are blue–brown macules occurring bilaterally on the upper portions of the face [95]. ABNOM is an acquired, bilateral condition thought to arise from the reactivation of dormant dermal melanocytes, potentially triggered by inflammation, skin aging, or an unknown cause [95]. HN is clinically similar to ABNOM but is often considered a distinct entity due to its later onset [10]. While these diagnoses are technically different, their treatment is very similar, and they are therefore often grouped together for analysis. Most patients enrolled with NOA, ABNOM, or HN had FST III or greater. Within this category, the most studied laser was again the Nd:YAG, followed by the Q-switched ruby and the Alexandrite PicoWay. Previously published reviews on the management of HN have outlined that laser treatment and dermabrasion are effective treatments in the management of this condition [10]. Similarly, NOA reviews have highlighted the efficacy of laser treatment in their management, especially with Q-switched lasers [7]. The most reported side effects were hyperpigmentation and erythema. As with CALMs, the hyperpigmentation is likely PIH in the setting of inflammation induced by the lasers. Additionally, erythema is a common side effect of nearly all laser treatments, especially appreciated in pulsed lasers, such as Q-switched lasers, due to epidermal immaturity and reduced melanin absorption of light [96].

Twenty studies were conducted on Becker’s nevus (BN), also known as Becker melanosis, with most studies enrolling patients with FSTs I through III. BN is a benign acquired hyperpigmentation with overlying hypertrichosis [97]. Due to the combination of hyperpigmentation and hypertrichosis, lasers are often utilized in the management of BN, which recent reviews have also highlighted [98]. This analysis, focusing solely on hyperpigmentation, found Nd:YAG to be the most studied laser, with the most common side effects being hyperpigmentation, erythema, and hypopigmentation.

There were twelve studies conducted on nevus spilus (NS), with most studies enrolling patients with FST I to III. Within the category of NS, there are many subtypes, but it generally presents as a hyperpigmented patch. An algorithm based on genetic and phenotypic patterns has been proposed for these subcategories [99]. While not applied in the studies reviewed, likely due to their publication before the algorithm was introduced, its inclusion in future studies could help elucidate if specific treatments are more efficacious for certain NS subtypes. The Nd:YAG was the most commonly studied treatment for NS, followed by the Alexandrite PicoWay. The most reported side effects of laser treatment for NS were hyperpigmentation, hypopigmentation, erythema, and pain.

While the search criteria included diagnoses such as dermal melanocytosis, Rhiel’s melanosis, nevus of Ito, lichen planus pigmentosus, erythema dyschromia perstans, erythromelanosis follicularis faciei, and linear and whorled nevoid hypermelanosis, there was a paucity of studies focusing on the utilization of lasers in their management. While these are rarer conditions, researchers should conduct additional studies to identify if preliminary success with laser management of these conditions can be replicated on a larger scale.

## 5. Conclusions

Within the realm of benign pigmented lesions, lasers are often a safe and efficacious choice for patients of all FSTs. However, a number of the studies did not report FST, which limits the generalizability of findings to diverse populations. FST is a critical determinant of laser–tissue interaction, particularly in relation to melanin content and the risk of pigmentary side effects. This omission underscores the need for more inclusive reporting standards to ensure that laser treatment recommendations are evidence-based and applicable to all skin types. Furthermore, we recommend that future studies not only include FST, but also utilize validated tools such as the Taylor Hyperpigmentation Scale and melanometers for objective measurements of skin pigmentation. Additionally, we acknowledge the potential for language bias introduced by the exclusion of non-English studies. Future reviews may benefit from multilingual search strategies or collaboration with translators to expand language inclusion criteria.

There is a paucity of data regarding cost of treatment and patient opinion on cost of laser treatment for pigmented lesions. Data regarding cost ought to also be published to identify treatments that are both efficacious and cost-effective for patients, especially as many lesions require multiple treatment sessions to reach partial or complete clearance. Despite the importance of affordability in treatment decision-making, only two studies in this review addressed cost. This omission is particularly relevant given that cosmetic laser treatments are often not reimbursed by insurance, potentially limiting access for many patients. Therefore, a thorough cost-effectiveness analyses ought to be included in future studies; not only for enhanced data on the subject matter, but also to ensure clinicians are able to counsel appropriately on cost-effectiveness of laser treatment for pigmented lesions.

Furthermore, patient preference ought to be studied concurrently to clinical clearance, as many patients may not seek full clinical clearance. Patient preference was inconsistently reported across studies, if reported at all. Future studies would benefit from a centralized patient preference reporting system, such as changes in the Dermatology Life Quality Index (DLQI) or the Patient Benefit Index (PBI), to elucidate the effect of these treatments not only on clinical clearance but also patient tolerability of these treatments. The addition of this data would be helpful in better understanding appropriate endpoints for the general patient population. Additionally, we did not differentiate whether “≥50% patient-reported clearance” was validated objectively (e.g., through clinician-rated photography) or subjectively via patient self-assessment, which may impact the reliability and comparability of reported outcomes across studies.

## Figures and Tables

**Table 1:** Efficacy of laser treatment in café-au-lait macules (CALMs): efficacy of laser treatment alongside FST distribution and side effect profile.

Laser	N (%)	FST	N (%)
**Nd:YAG**	17 (48.57%)	*I*	6 (17.14%)
*Successful*	15 (88.23%)	*II*	9 (25.71%)
*Not Successful*	2 (11.76%)	*III*	13 (37.14%)
**Q-Switched Ruby**	6 (17.14)	*IV*	15 (42.86%)
*Successful*	6 (100%)	*V*	4 (11.43%)
*Not Successful*	0 (0%)	*VI*	2 (5.71%)
**Alexandrite PicoWay**	6 (17.14%)	*FST not Documented*	18 (51.43%)
*Successful*	5 (83.33%)	**Side Effects**	**N (%)**
*Not Successful*	1 (16.67%)	*Hyperpigmentation*	15 (42.86%)
**Q-Switched Alexandrite**	5 (14.29%)	*Hypopigmentation*	12 (34.43%)
*Successful*	5 (100%)	*Erythema*	9 (25.71%)
*Not Successful*	0 (0%)	*Crusting*	7 (20%)
**PDL**	3 (8.57%)	*Scaring*	5 (14.29%)
*Successful*	3 (100%)	*Pain*	5 (14.29%)
*Not Successful*	0 (0%)	*Blistering*	5 (14.29%)
**IPL**	2 (5.71%)	*Edema*	5 (14.29%)
*Successful*	1 (50%)	*Depigmentation*	2 (5.71%)
*Not Successful*	1 (50%)	*Uneven pigmentation*	2 (5.71%)
**Copper Vapor**	1 (2.86%)	*Textural Changes*	1 (2.86%)
*Successful*	1 (100%)	*Textural Changes*	1 (2.86%)
*Not Successful*	0 (0%)		
**Erbium:YAG**	1 (2.86%)		
*Successful*	1 (100%)		
*Not Successful*	0 (0%)		
**Monoline Argon**	1 (2.86%)		
*Successful*	1 (100%)		
*Not Successful*	0 (0%)		
**Fractional**	1 (2.86%)		
*Successful*	1 (100%)		
*Not Successful*	0 (0%)		

**Note:** Laser treatment was classified as successful if the study reported statistically significant improvement or if more than 50% of treated patients demonstrated clinical improvement.

**Table 2:** Efficacy of laser treatment in nevus of Ota (NOA), acquired bilateral nevus of Ota-like macules (ABNOM), or Hori’s nevus: efficacy of laser treatment alongside FST distribution and side effect profile.

Laser	N (%)	FST	N (%)
**Nd:YAG**	**12 (41.14%)**	*I*	2 (6.90%)
*Successful*	11 (91.67%)	*II*	4 (13.79%)
*Not Successful*	1 (8.33%)	*III*	11 (37.93%)
**Q-Switched Ruby**	**9 (31.03%)**	*IV*	16 (55.17%)
*Successful*	8 (88.89%)	*V*	10 (34.48%)
*Not Successful*	1 (11.11%)	*VI*	4 (13.79%)
**Alexandrite PicoWay**	**7 (24.14%)**	*FST not Documented*	13 (44.83%)
*Successful*	6 (85.57%)	**Side Effects**	**N (%)**
*Not Successful*	1 (14.29%)	*Hyperpigmentation*	13 (44.83%)
**Q-Switched Alex**	**6 (20.69%)**	*Erythema*	10 (34.48%)
*Successful*	6 (100%)	*Hypopigmentation*	9 (31.03%)
*Not Successful*	0 (0%)	*Pain*	7 (24.14%)
**CO_2_**	**1 (3.45%)**	*Edema*	6 (20.69%)
*Successful*	1 (100%)	*Crusting*	6 (20.69%)
*Not Successful*	0 (0%)	*Depigmentation*	3 (10.34%)
**Argon**	**1 (3.45%)**	*Blistering*	3 (10.34%)
*Successful*	1 (100%)	*Petechiae*	3 (10.34%)
*Not Successful*	0 (0%)	*Superficial erosions*	2 (6.90%)
		*Punctate Hemorrhage*	2 (6.90%)

**Note:** Laser treatment was classified as successful if the study reported statistically significant improvement or if more than 50% of treated patients demonstrated clinical improvement.

**Table 3:** Efficacy of laser treatment in Becker’s nevus (BN): efficacy of laser treatment alongside FST distribution and side effect profile.

Laser	N (%)	FST	N (%)
**Nd:YAG**	**5 (25%)**	*I*	1 (5%)
*Successful*	5 (100%)	*II*	4 (20%)
*Not Successful*	0 (0%)	*III*	9 (45%)
**IPL**	**4 (20%)**	*IV*	10 (50%
*Successful*	3 (75%)	*V*	4 (20%)
*Not Successful*	1 (25%)	*VI*	1 (5%)
**Q-Switched Alex**	**3 (15%)**	*FST not Documented*	9 (45%)
*Successful*	1 (33.33%)	**Side Effects**	**N (%)**
*Not Successful*	2 (66.67%)	*Hyperpigmentation*	9 (45%)
**Erbium:YAG**	**3 (15%)**	*Erythema*	9 (45%)
*Successful*	3 (100%)	*Hypopigmentation*	7 (35%)
*Not Successful*	0 (0%)	*Crusting*	4 (20%)
**Q-Switched Ruby**	**2 (10%)**	*Scaring*	4 (20%)
*Successful*	2 (100%)	*Blistering*	4 (20%)
*Not Successful*	0 (0%)	*Pain*	3 (15%)
**Long Pulse Alex**	**2 (10%)**	*Edema*	3 (15%)
*Successful*	1 (50%)	*Textural changes*	3 (15%)
*Not Successful*	1 (50%)	*Folliculitis*	1 (5%)
**Alexandrite PicoWay**	**2 (10%)**	*Depigmentation*	1 (5%)
*Successful*	2 (100)	*Uneven pigmentation*	1 (5%)
*Not Successful*	0 (0%)		
**Thulium**	**1 (5%)**		
*Successful*	1 (100%)		
*Not Successful*	0 (0%)		
**Argon**	**1 (5%)**		
*Successful*	0 (0%)		
*Not Successful*	1 (100%)		
**Monoline Argon**	**1 (5%)**		
*Successful*	1 (100%)		
*Not Successful*	0 (0%)		
**Fractional**	**1 (5%)**		
*Successful*	1 (100%)		
*Not Successful*	0 (0%)		

**Note:** Laser treatment was classified as successful if the study reported statistically significant improvement or if more than 50% of treated patients demonstrated clinical improvement.

**Table 4:** Efficacy of laser treatment in nevus spilus (NS): efficacy of laser treatment alongside FST distribution and side effect profile.

Laser	N (%)	FST	N (%)
**Nd:YAG**	**5 (41.67%)**	*I*	2 (16.67%)
*Successful*	3 (60%)	*II*	3 (25%)
*Not Successful*	2 (40%)	*III*	4 (33.33%)
**Pico Alex**	**3 (25%)**	*IV*	4 (33.33%)
*Successful*	3 (100%)	*V*	1 (8.33%)
*Not Successful*	0 (0%)	*VI*	0 (0%)
**Monoline Argon**	**2 (16.67%)**	*FST not Documented*	7 (58.33%)
*Successful*	2 (100%)	**Side Effects**	**N (%)**
*Not Successful*	0 (0%)	*Hyperpigmentation*	5 (41.67%)
**Q-Switched Ruby**	**2 (16.67%)**	*Hypopigmentation*	4 (33.33%)
*Successful*	1 (50%)	*Erythema*	4 (33.33%)
*Not Successful*	1 (50%)	*Pain*	4 (33.33%)
**IPL**	**2 (16.67%)**	*Blistering*	3 (25%)
*Successful*	2 (100%)	*Crusting*	3 (25%)
*Not Successful*	0 (0%)	*Scaring*	2 (16.67%)
**CO_2_**	**2 (16.67%)**	*Depigmentation*	1 (8.33%)
*Successful*	2 (100%)	*Uneven pigmentation*	1 (8.33%)
*Not Successful*	0 (0%)	*Edema*	1 (8.33%)
**Argon**	**1 (8.33%)**		
*Successful*	1 (100%)		
*Not Successful*	0 (0%)		
**Q-Switched Alex**	**1 (8.33%)**		
*Successful*	1 (100%)		
*Not Successful*	0 (0%)		

**Note:** Laser treatment was classified as successful if the study reported statistically significant improvement or if more than 50% of treated patients demonstrated clinical improvement.

## Abbreviations

The following abbreviations are used in this manuscript:

CALMs	Café-au-lait macules
NOA	Nevus of Ota
BN	Becker’s nevus
LPP	Lichen planus pigmentosus
Nd:YAG	Q-switched neodymium-doped yttrium aluminum garnet
PIH	Post-inflammatory hyperpigmentation
PRISMA	Preferred Reporting Items for Systematic Reviews and Meta-Analyses
FST	Fitzpatrick skin type
ABNOM	Acquired bilateral nevus of Ota-like macules
IPL	Intense pulsed light
NS	Nevus spilus
ECP	Erythema dyschromia perstans
EFF	Erythromelanosis follicularis faciei
LWNH	Linear and whorled nevoid hypermelanosis
HN	Hori’s nevus
DLQI	Dermatology Life Quality Index
PBI	Patient Benefit Index
